# Immunophenotypic characteristics and prognostic value of peripheral blood circulating plasma cells in patients with newly diagnosed multiple myeloma

**DOI:** 10.5937/jomb0-55319

**Published:** 2025-06-13

**Authors:** Hong Chen, Yuan Zhao, Zhiyu Zhang, Yan Xie, Mulan Jin

**Affiliations:** 1 Capital Medical University, Beijing Chaoyang Hospital, Department of Pathology, Beijing, China

**Keywords:** multiple myeloma, circulating plasma cells, immunophenotyping, flow cytometry, prognosis, multipli mijelom, cirkulišuće plazma ćelije, imunofenotipizacija, protočna citometrija, prognoza

## Abstract

**Background:**

To investigate the immunophenotypic characteristics and prognostic value of peripheral blood circulating plasma cells (CPCs) in patients with newly diagnosed multiple myeloma (NDMM).

**Methods:**

This retrospective study was conducted on NDMM patients treated at Beijing Chaoyang Hospital, Capital Medical University, between January 2020 and June 2023. A total of 57 patients were included, with a median age of 64 years, comprising 27 males. Forty-four patients were assigned to the higher CPCs group and 13 to the lower CPCs group. To compare the proportion of bone marrow plasma cells (BMPCs) between the two groups and to analyse the differences in the immunophenotypes of BMPCs and CPCs. Subsequently, the prognosis of the patients was analysed by COX.

**Results:**

The percentage of BMPCs was significantly higher in the higher CPCs group compared to the lower CPCs group (53.07% vs. 15.23%, P<0.001). In the higher CPCs group, BMPCs exhibited decreased expression of CD56 and CD27 but increased expression of CD81 (all P<0.05). The median PFS in the lower CPCs group (17.6 months, 9.12-31.54) was significantly higher than that in the higher CPCs group (14.1 months, 5.08-26.12) (P=0.015). Multivariate Cox regression analysis identified CPCs ≥ 0.0101% (HR=6.721, 95% CI: 3.891-11.224, P<0.001) as the independent prognostic factors for PFS.

**Conclusions:**

This study demonstrates distinct immunophenotypic differences between the higher and lower CPCs groups in NDMM patients.

## Abstract (sr)

**Uvod:**

Cilj je bio da se istraže imunofenotipske karakteristike i prognostička vrednost cirkulišućih plazma ćelija (CPC) u perifernoj krvi kod pacijenata sa novodijagnostikovanim multiplim mijelomom (NDMM).

**Metode:**

Ova retrospektivna studija obuhvatila je NDMM pacijente lečene u Pekinškoj bolnici Chaoyang, Medicinskog fakulteta u glavnom gradu, izme|u januara 2020. i juna 2023. godine. Uključeno je ukupno 57 pacijenata, sa medianom starosti od 64 godine, od kojih 27 muškaraca. Četrdeset četiri pacijenta su svrstana u grupu sa višim nivoom CPC, dok je 13 pacijenata bilo u grupi sa nižim nivoom CPC. Upoređivan je procenat plazma ćelija u koštanoj srži (BMPC) između dve grupe, kao i razlike u imunofenotipu BMPC i CPC. Prognoza pacijenata analizirana je korišćenjem COX modela.

**Rezultati:**

Procenat BMPC bio je značajno viši u grupi sa višim nivoom CPC u poređenju sa grupom sa nižim CPC (53,07% naspram 15,23%, P<0,001). U grupi sa višim nivoom CPC, BMPC su pokazale smanjenu ekspresiju CD56 i CD27, ali povećanu ekspresiju CD81 (sve P<0,05). Medijana preživljavanja bez progresije bolesti (PFS) bila je značajno viša u grupi sa nižim CPC (17,6 meseci, 9,12-31,54) u poređenju sa grupom sa višim CPC (14,1 mesec, 5,08-26,12) (P=0,015). Multivarijantna Cox regresiona analiza identifikovala je nivo CPC ≥0,0101% (HR=6,721, 95% CI: 3,891-11,224, P<0,001) kao nezavisni prognostički faktor za PFS.

**Zaključak:**

Ova studija ukazuje na postojanje specifičnih imunofenotipskih razlika između pacijenata sa višim i nižim nivoom CPC u NDMM.

## Introduction

Multiple myeloma (MM) is a malignant plasma cell tumour predominantly affecting individuals over 60. It is the second most common haematological malignancy, accounting for approximately 10% of all haematological malignancies [1]. It is characterised by abnormal proliferation of bone marrow plasma cells (BMPCs) and excessive monoclonal immunoglobulin or light chains (M protein). It is often associated with multiple osteolytic lesions, hypercalcemia, anaemia, and renal impairment [2] [3]. Traditional diagnostic methods for MM require assessing abnormal protein levels in the serum or urine, imaging studies of the entire skeletal system, and bone marrow biopsies to evaluate the proportion of abnormal plasma cells in the bone marrow. This is because clonal plasma cells predominantly reside in the bone marrow, adhering to the microenvironment [4] [5]. As such, bone marrow aspiration is also an important traditional method for MM that provides rapid diagnosis, therapeutic response monitoring, and prognostic evaluation [6]; however, this procedure is often a painful experience for patients. Additionally, due to bone marrow dilution and the focal distribution of tumour cells, bone marrow aspiration or a single bone marrow biopsy may not reflect the genetic heterogeneity of MM [6]. An analysis involving multiple regions of the iliac bone confirmed that over 75% of MM patients exhibited spatial genomic heterogeneity [7].

Immunotyping plasma cells has become an important part of the diagnosis process. It can help identify and distinguish malignant plasma cells from normal cells by identifying aberrant expression of CD19 and other markers [8]. Immunotyping can also help predict prognosis; for example, cases with newly diagnosed MM (NDMM) cases with a proportion of more than 5% normal plasma cells from bone marrow have better prognosis than patients with less than 5% normal plasma cells [9]. As MM progresses, malig nant plasma cells can migrate from the bone marrow into peripheral blood (PB), forming circulating plasma cells (CPCs) [10]. CPCs are one of the factors contributing to distant dissemination, recurrence with drug resistance, and the presence of extra medullary disease in MM [10]. Accordingly, CPCs can be of value in the early diagnosis of MM, therapeutic response monitoring, and prognostic evaluation [11] [12].

Detecting CPCs via multiparameter flow cytometry (MFC) can eliminate the need for repeated invasive bone marrow biopsies and offers higher sensitivity than traditional histopathological techniques [13]. However, the optimal threshold of CPCs varies across populations, typically ranging from 10 CPCs/50,000 nucleated cells to 400 CPCs/150,000 nucleated cells [14] [15] [16] [17] [18] [19].

Currently, there are limited studies in China examining the correlation between the proportion of BMPCs and CPCs; only a few studies have suggested a potential link between CPCs and prognosis in MM [20] [21] [22]. Meanwhile, it is also unclear whether morphological relationships and immunophenotypic similarities and differences exist between BMPCs and CPCs in NDMM patients. Therefore, studies are needed to provide information on these aspects of CPCs for Chinese populations. This study aimed to examine the immunophenotypic characteristics of CPCs and BMPCs in newly diagnosed MM patients and to evaluate the prognostic significance of CPCs in this population. These findings will provide new references and guidelines for the future diagnosis and treatment of MM.

## Materials and methods

### Study design and patients

This retrospective study included patients diagnosed with NDMM who attended the Department of Hematology at the Shijingshan District of Beijing Chaoyang Hospital, Capital Medical University, between January 2020 and June 2023. We calculated the required sample size for this study by using GPower software and subsequently included 57 study subjects according to the inclusion-exclusion criteria. The inclusion criteria were: 1) Patients who met the diagnostic criteria for MM as outlined in the Chinese Guidelines for the Diagnosis and Treatment of MM (Revised in 2020) [23]. 2) Patients aged >18 years. 3) complete dataset. The exclusion criteria were: 1) Patients with other types of malignant haematological diseases. 2) Patients with infectious or immunological diseases. 3) Patients with other types of malignant tumours. 4) Patients with organ failure.

A total of 57 patients with NDMM were included in this study, consisting of 27 males and 30 females aged 44 to 90 years, with a median age of 64 years. Among these, 26 cases were classified as IgG, 14 as IgA, 1 as IgD, and 16 as light chain myeloma. Based on the DS staging system, 5 patients were in stages I+II, and 52 were in stage III. According to the R-ISS, 14 patients were in stages I+II, while 43 were in stage III. We categorised patients into two groups based on their median CPCs of 0.0101%: the higher CPCs group (CPCs≥0.0101%, n=44) and the lower CPCs group (CPCs<0.0101%, n=13).

**Figure 1:**
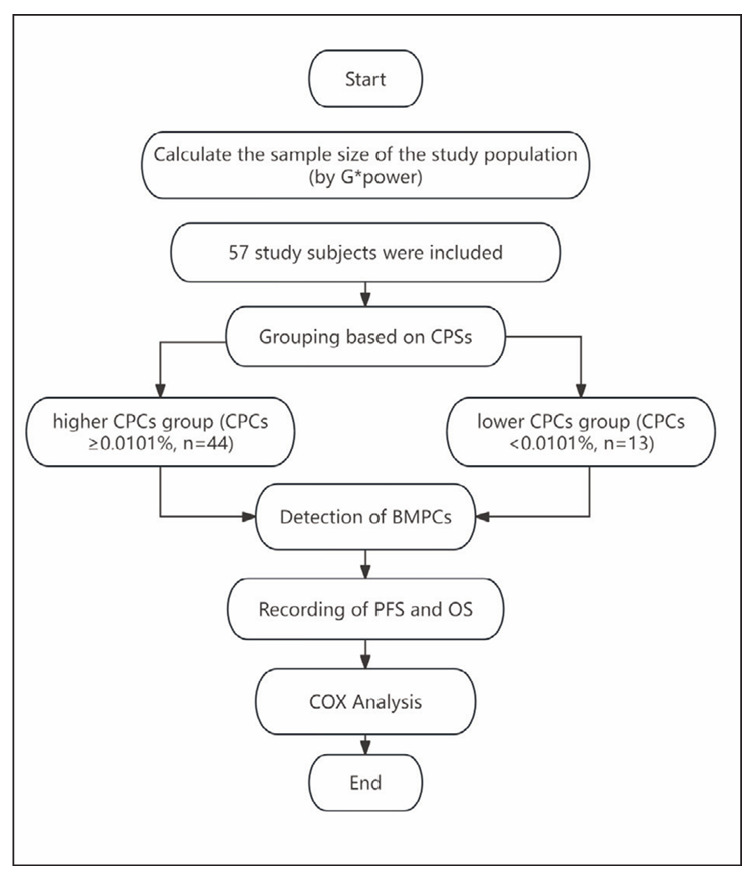
Main flow of this study.

All patients underwent BM biopsy, flow cytometry analysis of both BM and PB. This study was approved by the Medical Research Ethics Committee of Beijing Chaoyang Hospital, Capital Medical University, and informed consent was waived for the retrospective nature of this study. Furthermore, we will conduct the study strictly following the Declaration of Helsinki. The main flow of this study is shown in Figure 1.

### Flow cytometry sample preparation and detection

PB and BM samples (2 mL each) were collected from patients using ethylenediaminetetraacetic acid (EDTA) as an anticoagulant. A 500 μL aliquot of BM or PB was mixed with ammonium chloride lysing reagent at a 1:10 ratio, vortexed thoroughly, and left to stand for 15 minutes. Subsequently, 5 mL of phosphate-buffered saline (PBS) was added, and the mixture was centrifuged at 1500 rpm for 5 minutes, with the supernatant discarded after each of the two washes. The washed cells (50–100 μL) were incubated with a panel of eight membrane antibodies (CD19-PerCP, CD56-PE-Cy7, CD138-APC, CD38-APC-H7, CD27-BV421, CD45-V500, CD81-BV605, and CD200-APC-R700) at room temperature for 15 minutes. The monoclonal fluorescent antibodies and isotype controls used in this study were all purchased from BD Biosciences (USA). A further 100 μL of Fixative A was added, and the cells were incubated in the dark for another 15 minutes. The cells were washed with 2 mL of PBS, centrifuged at 1500 rpm for 5 minutes, and the supernatant was discarded. After resuspending the pellet, 100 μL of Permea bilization Reagent B was added, and the mixture was gently pipetted to ensure even suspension. Cyto plasmic antibodies (cKappa-FITC and cLambda-PE) were added, and the cells were incubated in the dark at room temperature for 30 minutes. After washing with 2 mL of PBS and centrifuging at 1500 rpm for 5 minutes, the supernatant was discarded, and the cell pellet was resuspended in 500 μL of PBS for flow cytometry analysis.

Flow cytometric analysis was performed using the BD FACS Canto flow cytometer (BD Biosciences, USA), and data analysis was conducted using BD FACS Diva software. The CD45/Time parameter was used to select a stable fluid stream, and cell aggregates were excluded based on FSC-H/FSC-A and SSC-H/SSC-A plots. Cell debris was then removed using SSC-A/FSC-A plots. A total of 100,000 nucleated cells were acquired from BM samples, and 1,000,000 nucleated cells were acquired from PB samples. CD38^+^CD138^+^ and CD38^bri^CD138^−^ cell populations were identified, and accurate plasma cell (PC) populations were gated using a combination of CD45 and SSC. The percentage of aberrant phenotypic monoclonal plasma cells in the total nucleated cells of BM and PB was recorded for each patient, with a detection sensitivity of 10^-5^.

### Cytogenetic analysis

Fluorescence in situ hybridisation (FISH) cytogenetic analysis was performed on BM samples from patients to detect abnormalities such as IgH translocation, 1q21 amplification, 17p- (P53 deletion), and 1p32 deletion. According to the Mayo revised mSMART 3.0 prognostic assessment system (www.mSMART.org), additional tests for t(4;14), t(14;16), and t(14;20) were conducted when IgH breakage was detected. Genetic high-risk was defined as the presence of 1q21 amplification, 17p- (P53 deletion), P53 mutation, t (4;14), t (14;16), or t (14;20), while patients without these abnormalities were classified as standard genetic risk [23]. The test reagents used in this study were obtained from Guangzhou Anbiping Pharmaceutical Technology Co., Ltd.

### Data collection

Data on demographics, such as gender and age, as well as classification of NDMM, Durie-Salmon (DS) staging, Revised International Staging System (R-ISS) staging, and laboratory results including haemoglobin (Hb, g/L), platelet count (PLT, *10^9^/L), albumin (ALB, g/L), lactate dehydrogenase (LDH, U/L), Ca (mmol/L), creatinine (Cr, μmol/L), and beta-2 macroglobulin (β2-MG, mg/L) were collected from the medical records.

Patient medical records were reviewed, and survival follow-up was conducted through telephone contact with patients or their relatives. The frequency of follow-up visits was 1 per month, and the follow-up period ended on 1st July 2023. The endpoints of this study were overall survival (OS) and progression-free survival (PFS). OS was defined as the time from diagnosis to death from any cause or last follow-up, while PFS was defined as the time from diagnosis to disease progression, death, or last follow-up.

### Statistical analysis

Statistical analyses were performed using SPSS 27.0 (IBM Corp., NY, USA). The normal distribution of continuous data was checked. The continuous variables conforming to the normal distribution were described as means ± standard deviations (SD) and analysed using Student’s t-test or analysis of variance (ANOVA). Those with a skewed distribution were presented as median (P25-P75) and analysed using the Wilcoxon rank-sum tests. The categorical variables were described as n (%) and were compared using X^2^ tests. Correlations were assessed using Spearman’s correlation analysis. Survival analysis was conducted using the Kaplan-Meier method to plot survival curves for OS and PFS in MM patients. The log-rank test was compared with survival curves of different influencing factors. Multivariate Cox proportional hazards regression analysis explored factors affecting OS and PFS in MM patients. A p-value <0.05 was considered statistically significant.

## Results

### Clinical and biological characteristics between the higher CPCs and lower CPCs groups

The levels of Hb (P=0.026), PLT (P=0.049), and ALB (P=0.033) in the higher CPCs group were lower than those in the lower CPCs group, while LDH (P=0.029) and β2-MG (P=0.043) levels were higher in the higher CPCs group. Additionally, significant differences were observed in R-ISS staging (P=0.040) and cytogenetic abnormalities (P=0.024) between the two groups (Table 1).

**Table 1:** Comparison of clinical and biological characteristics between the higher CPCs and lower CPCs groups. Abbreviations: CPCs, circulating plasma cells; Ig, immunoglobulin; Hb, haemoglobin; PLT, platelet count; ALB, albumin; LDH, lactate dehydrogenase; Cr, creatinine; β2-MG, beta-2 macroglobulin; DS, Durie-Salmon; R-ISS, Revised International Staging System.

	Higher CPCs Group (N=44)	Lower CPCs Group (N=13)	t or χ^2^ Values	P Values
Gender (Male, %)	22 (50.0%)	5 (38.5%)	0.536	0.464
Age (Median, Range)	64.8 (44–90)	65.3 (52–80)	0.741	0.882
Classification (N, %)				
IgG Type	22 (50.0%)	4 (30.8%)	2.021	0.473
IgA Type	10 (22.7%)	4 (30.8%)		
Light Chain Type	11 (25%)	5 (38.4%)		
Other	1 (2.3)	0 (0.0%)		
Laboratory Tests				
Hb (g/L)	90.5 (52–151)	109.1 (55–149)	5.364	0.026
PLT (*10^9^/L)	132.0 (8–328)	185.7 (11–340)	3.642	0.049
ALB (g/L)	32.0 (22.4–45.8)	36.85 (25.0–42.7)	4.167	0.033
LDH (U/L)	275.94 (120.2–719.4)	184.78 (146.7–267.7)	5.284	0.029
Ca (mmol/L)	2.23 (1.5–4.07)	2.18 (1.7–2.48)	1.720	0.665
Cr (μmol/L)	134.34 (29.5–625.6)	97.32 (32.3–370.8)	2.116	0.273
β2-MG (mg/L)	7.01 (1.47–33.58)	3.28 (1.29–9.36)	3.684	0.043
DS Staging (N, %)				
Stages I + II	2(4.5%)	3(23.1%)	3.306	0.072
Stage III	42(95.5%)	10(76.9%)		
R-ISS Staging (N, %)				
Stages I + II	8 (18.2%)	6 (46.2%)	4.238	0.040
Stage III	36 (81.8%)	7 (53.8%)		
Cytogenetics (N, %)				
High Risk	24 (54.5%)	2 (15.4%)	6.204	0.024
Standard Risk	20 (45.5%)	11 (84.6%)		

### Morphology of plasma cells in bone marrow (BM) biopsy

**Figure 2:**
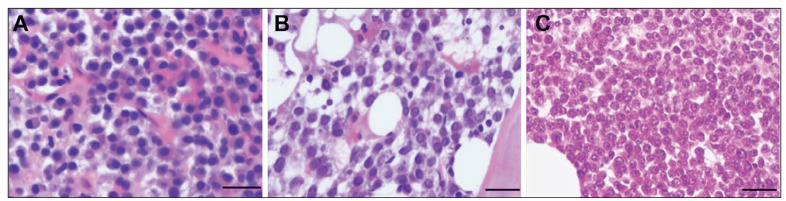
(A) Mature plasma cells diffusely distributed, with nuclear displacement and indistinct nucleoli (scale: 20 μm). (B) Immature plasma cells show a nodular distribution, with enlarged nuclei and prominent nucleoli (scale: 20 μm). (C) Plasma blast-like plasma cells are diffusely distributed, with large nuclei, central nucleoli, and dense chromatin (scale: 20 μm).

Histological examination of BM biopsy samples revealed scattered, clustered, or sheet-like distribution of plasma cells within the trabecular spaces, with some exhibiting diffuse infiltration. Plasma cells displayed eccentric nuclei with inconspicuous nucleoli; some cells appeared enlarged with centrally located nuclei and prominent nucleoli, exhibiting atypical features (Figure 2).

### Morphology, proliferation patterns, and immunophenotypes of plasma cells in the bone marrow of NDMM patients

In the higher CPCs group, the proportion of BMPCs was 53.07%, with the patterns of plasma cell proliferation being: nodular type (23 cases, 52.3%), interstitial type (15 cases, 34.1%), and diffuse type (6 cases, 13.6%). In the lower CPCs group, the proportion of BMPCs was 15.23%, with the interstitial type being the most common (8 cases, 61.5%), followed by the nodular type (5 cases, 38.5%), and no diffuse type was observed. Regarding the morphology of BMPCs, both groups primarily consisted of mature plasma cells. The proportion of BMPCs in the higher CPCs group was significantly higher than that in the lower CPCs group (P<0.001). However, the two groups observed no significant differences in plasma cell morphology or proliferation patterns.

A comparison of BMPC of flow cytometry between the two groups revealed that the median percentage of BMPCs in the higher CPCs group was 12.3%, significantly greater than the 0.9% observed in the lower CPCs group (P<0.05). Regarding immune phenotypes, CD56 (P<0.001) and CD27 (P<0.001) expression in BMPCs were significantly lower in the higher CPCs group compared to the lower CPCs group, while CD81 expression was notably higher in the higher CPCs group (P<0.001) (Table 2).

**Table 2:** Morphology, proliferation patterns, and immunophenotypes of plasma cells in the bone marrow of NDMM patients. Abbreviations: CPCs, circulating plasma cells; BM, bone marrow; BMPC, bone marrow plasma cells.

	Higher CPCs Group<br>(N=44)	Lower CPCs Group<br>(N=13)	t or χ^2^ Values	P Values
BMPC of BM biopsy (Median %, Range %)	53.07 (10–90)	15.23 (3–40)	8.467	<0.001
Plasma Cell Proliferation Patterns (Number of Cases, %)			4.036	0.133
Stromal Type	15 (34.1%)	8 (61.5%)		
Nodular Type	23 (52.3%)	5 (38.5%)		
Diffuse Type	6 (13.6%)	0 (0%)		
Plasma Cell Morphology (Number of Cases, %)			0.829	0.948
Mature Type	25 (56.8%)	8 (61.5%)		
Immature Type	5 (11.4%)	2 (15.4%)		
Progenitor Type	2 (4.5%)	0 (0.0%)		
Intermediate Type	12 (27.3%)	3 (23.1%)		
Immunophenotype analysis of BM biopsy				
BMPC of BM flow cytometry (Median %, Range %)	12.3 (0.19–50.0)	0.9 (0–5.88)	5.942	0.001
CD38 (Number of Cases, %)	44 (100%)	13 (100%)	>0.999	>0.999
CD138	44 (100%)	13 (100%)	>0.999	>0.999
CD19	30 (68.18%)	10 (76.92%)	0.366	0.795
CD56	7 (15.91%)	11 (85.62%)	21.920	<0.001
CD27	4 (9.09%)	10 (76.92%)	24.923	<0.001
CD200	35 (79.55%)	8 (61.54%)	1.756	0.185
CD81	34 (77.27%)	1 (7.69%)	20.503	<0.001

### Consistency analysis of immune phenotypes and clonality between BMPCs and CPCs

**Figure 3:**
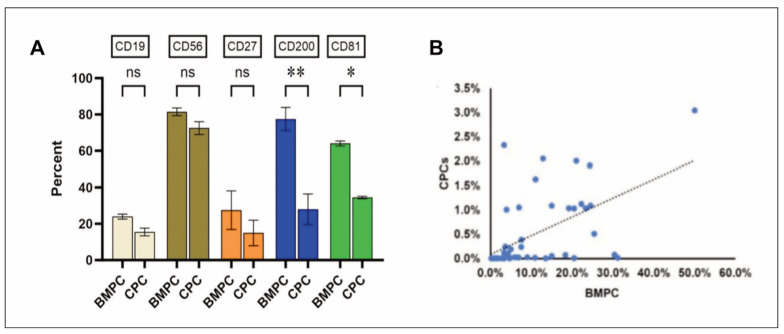
(A) Immunophenotypic comparison between bone marrow plasma cells (BMPCs) and circulating plasma cells (CPCs). ns P>0.05, *P<0.05, **P<0.01. (B) Positive correlation between the percentages of circulating plasma cells (CPCs) and bone marrow plasma cells (BMPCs).

All 57 patients (100.0%) exhibited intracellular light chain restriction/monoclonal expression, with a cKappa expression rate of 43.0% and a cLambda expression rate of 57.0%. 44 NDMM patients (77.2%) displayed CPCs, all demonstrating intracellular light chain restriction/monoclonal expression, with a cKappa expression rate of 49.0% and a cLambda expression rate of 51.0%. The abnormal plasma cells in BMPCs compared to CPCs in the same patient exhibited homogeneity in the immune phenotype. Compared to BMPCs, CPCs showed decreased expression of CD200 and CD81 (P<0.05), while there were no significant differences in the expression of CD19, CD56, and CD27, and clonality was consistent (Figure 3A). Spearman correlation analysis indicated a positive correlation between the percentage of CPCs and BMPCs (r=0.561, P<0.001) (Figure 3B).

### The relationship between the prognosis of CPCs

**Figure 4:**
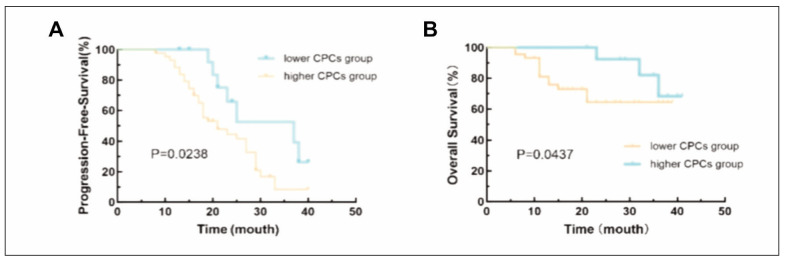
(A) Kaplan-Meier survival curve for progression-free survival (PFS) between the higher CPCs and lower CPCs groups. (B) Kaplan-Meier survival curve for overall survival (OS) between the higher CPCs and lower CPCs groups.

Among the 57 NDMM patients, the median PFS in the higher CPCs group was 21 months, while the median PFS in the lower CPCs group was 37 months. The difference was statistically significant (P=0.024) (Figure 4A). The median OS in the higher CPCs group and lower CPCs group was not reached, and the difference was statistically significant (P=0.044) (Figure 4B).

### COX analysis affecting PFS and OS in NDMM patients

The variables assessed included sex, age, Hb, ALB, LDH, Ca, Cr, β2-MG, DS stage, R-ISS stage, High-Risk Cytogenetics, BMCP and CPCs. In the univariate model, LDH, β2-MG, DS stage, R-ISS stage, high-risk cytogenetics, BMCP and CPCs were found to affect PFS. DS stage, high-risk cytogenetics and CPCs were found to affect OS signi cantly. Further multivariate analysis using patient survival as the independent variable showed that LDH, DS stage, high-risk cytogenetics and CPCs were associated with PFS. Similarly, high-risk cytogenetics and CPCs were signi cantly related to OS (Table 3).

**Table 3:** Univariate and multivariate analyses of factors influencing PFS and OS in NDMM patients. Abbreviations: HR, hazard ratio; CI, confidence interval; CPCs, circulating plasma cells; Ig, immunoglobulin; Hb, haemoglobin; PLT, platelet count; ALB, albumin; LDH, lactate dehydrogenase; Cr, creatinine; β2-MG, beta-2 macroglobulin; DS, Durie-Salmon; R-ISS, Revised International Staging System; BMPC, bone marrow plasma cells.

	PFS				OS			
Factors	Univariate analysis		Multivariate analysis		Univariate analysis		Multivariate analysis	
	* HR(95%CI) *	* P *	* HR(95%CI) *	* P *	* HR(95%CI) *	* P *	* HR(95%CI) *	* P *
Sex<br>(Male vs Female)	0.898<br>(0.524–1.467)	0.634			1.231<br>(0.982–2.121)	0.216		
Age≥65 years	1.006<br>(0.965–1.049)	0.778			1.382<br>(0.783–2.492)	0.321		
Hb 100 g/L	0.529<br>(0.272–1.029)	0.061			0.891<br>(0.472–1.321)	0.051		
ALB<35 g/L	1.782<br>(0.672–3.514)	0.350			2.133<br>(0.873–4.238)	0.193		
LDH≥220 U/L	1.704<br>(1.257–2.843)	0.023	1.463<br>(1.003–2.189)	0.041	2.331<br>(1.342–4.839)	0.012	1.345<br>(0.923–1.832)	0.068
Ca>2.65 mmol/L	2.415<br>(0.981–5.124)	0.056			1.232<br>(0.843–2.537)	0.318		
Cr≥177 μmol/L	1.325<br>(0.782–2.742)	0.072			1.453<br>(0.649–3.129)	0.672		
β2-MG≥3.5 mg/L	2.345<br>(1.628–4.982)	0.019	1.774<br>(0.923–3.136)	0.225	2.421<br>(0.891–4.782)	0.381		
DS Stage III	3.735<br>(1.056–8.206)	0.008	3.132<br>(1.039–7.385)	0.021	4.321<br>(1.324–7.342)	0.034	3.214<br>(1.292–6.432)	0.056
R-ISS Stage III	2.992<br>(1.246–8.181)	0.014	2.374<br>(0.927–6.237)	0.067	2.839<br>(0.932–4.516)	0.056		
High-Risk<br>Cytogenetics	3.002<br>(1.782–5.409)	<0.001	2.671<br>(1.671–4.889)	0.004	4.219<br>(1.782–7.389)	<0.001	3.810<br>(1.572–6.892)	0.012
BMCP≥30%	3.232<br>(1.292–7.428)	0.039	2.772<br>(0.832–5.231)	0.178	2.412<br>(0.781–4.214)	0.267		
CPCs≥0.0101%	5.548<br>(3.218–10.539)	<0.001	5.021<br>(2.328–8.323)	<0.001	5.289<br>(2.894–9.148)	0.002	4.249<br>(1.894–8.781)	0.043

## Discussion

The findings of this study demonstrated that BMPCs were elevated in the higher CPCs group; however, plasma cell morphology and proliferative patterns remained consistent within the same individual. Patients with higher CPCs had a poorer prognosis. Additionally, multivariate analysis identified LDH220 U/L, DS stage III, high-risk cytogenetic abnormalities, and CPCs0.0101% were clearly associated with PFS. Similarly, high-risk cytogenetics and CPCs≥0.0101% were significantly related to OS.

The findings of this study revealed that patients in the higher CPCs group exhibited significantly lower levels of Hb, PLT, and ALB compared to the lower CPCs group, whereas LDH and β2-MG levels were markedly higher. LDH and β2-MG are critical indicators for evaluating the disease status, therapeutic efficacy, and prognosis of MM, as serum levels of LDH and β2-MG are often proportional to the total number of MM cells [24]. Additionally, this study observed significant differences in R-ISS staging and cytogenetic abnormalities, with a higher R-ISS stage and a greater proportion of high-risk cytogenetic abnormalities in the higher CPCs group. Thus, patients in the higher CPCs group exhibit poorer prognoses regarding renal function impairment, tumour cell activity, and risk stratification.

There have been limited reports on the correlation between CPCs and BMPC morphology and immunophenotype. One study reported a non-linear correlation between the absolute count of CPCs and the BMPC percentage when using MFC for CPC enumeration [25]. This study verified a positive correlation between CPCs and BMPCs using the MFC method (r=0.561, P=0.000). Therefore, MFC detection of CPCs can be used to monitor tumour burden in the bone marrow during treatment without the need for repeated bone marrow biopsies. Based on pathological observations of bone marrow biopsy specimens, our study found that the proportion of BMPCs in the higher CPCs group was significantly higher than that in, the lower CPCs group. Nodular infiltration of BMPCs was more common in the higher CPCs group, whereas stromal infiltration was more prevalent in the lower CPCs group. In both groups, the morphology of plasma cells was mainly mature, accounting for 56.8% and 61.5%, respectively, with the least proportion of immature plasma cells (plasma blasts). However, the two groups had no significant differences in plasma cell proliferation patterns and morphology. We speculate that this may be due to the small number of specimens in each group. No diffuse infiltration or plasma blast morphology was observed in the lower CPCs group, which may not accurately represent the overall disease condition. This highlights the need for future studies with larger sample sizes to confirm the relationship between the two.

Using MFC, we found that the phenotype and clonality of CPCs were essentially consistent with those of BMPCs in the same patient. This finding is consistent with the results of Paiva et al. [26]. CPCs exhibited the same phenotype as their paired BMPCs, with both displaying similar expression levels of B-cell maturation-associated markers, such as CD19, CD20, CD45, and CD79b [26]. A previous study indicated that, compared to BMPCs, CPCs exhibit lower levels of integrins (e.g., CD11a, CD11c, CD29, CD49d, CD49e), CD33, adhesion molecules (CD56), and stem cell factor receptors (CD117) [26]. Although our study was limited in terms of detection indicators and did not observe differences in the expression of these factors, our findings revealed that BMPCs in the higher CPCs group had lower expression of CD56 and CD27 and higher expression of CD81 compared with the lower CPCs group. These phenotypic changes facilitate the migration of BMPCs into PB to form CPCs and are associated with lower proliferative capacity. Furthermore, whole-exome sequencing analysis confirmed high consistency between CPCs and bone marrow samples regarding clonal changes at different disease stages. However, certain dominant CPCs exhibited sub-clonal alterations [27] [28]. Thus, next-generation sequencing combined with high-purity fluorescence-activated cell sorting based on patient-specific aberrant phenotypes, or single-cell RNA sequencing, may provide a more accurate method for identifying MM cell characteristics.

In this study, we also observed that a small number of immunophenotypically normal polyclonal plasma cells were present in the PB of certain MM patients alongside monoclonal tumour plasma cells (CPCs). Compared with normal plasma cells, CPCs exhibited lower CD45, CD38, CD27, and CD81 expression, typically lacked CD19 expression, and expressed CD56. These features enable the differentiation between minute quantities of normal and abnormal plasma cells, thereby avoiding the omission of minor clonal cell populations. Our comparison revealed that the phenotype of the small number of normal plasma cells in the PB of these patients was largely consistent with that of normal plasma cells in the bone marrow.

In this population of NDMM patients, LDH levels ≥220 U/L, stage III in the DS staging system, high-risk cytogenetic, and CPCs ≥0.0101% were all independent adverse prognostic factors influencing PFS in NDMM patients. Meanwhile, high-risk cytogenetic and CPCs ≥0.0101% were independent adverse prognostic factors influencing OS. This finding provides robust evidence, beyond laboratory and molecular examinations, for the prognostic evaluation of NDMM patients in routine clinical practice. Moreover, CPCs, in combination with PET-CT can enhance the capability for risk stratification [17] and optimise the risk stratification effect of the R-ISS model [29], which has already been adopted in the expert consensus in China [30].

Bone marrow examination remains the gold standard for prognostic evaluation and MRD detection in most MM patients. This study supports the view that liquid biopsy to determine CPCs can serve as an effective clinical testing method due to its noninvasive, painless, accurate, and reliable characteristics. Nevertheless, this technology requires validation and support through extensive clinical trials before being applied in clinical practice. One essential condition is to integrate these findings into the management strategies of MM patients, providing clear evidence of full consistency with traditional goldstan dard parameters, including M-protein levels, bone marrow aspiration, and imaging examinations. It is anticipated that CPCs will soon be standardised as a widely available indicator for precise prognostic evaluation and risk stratification in MM.

As a retrospective analysis in a single centre, the study size was limited, and there may have been some bias in including the patients. The analysis of antigens was also limited, and we did not perform any sequencing, so the full complexity of the CPC populations was not investigated. Further prospective studies in larger sample sizes from multiple centres are needed.

## Conclusion

The results of this study indicate distinct immunophenotypic differences among NDMM patients with varying levels of CPCs. In the higher CPCs group, the expression of CD56 and CD27 in BMPCs was significantly lower compared to the lower CPCs group, while CD81 expression was higher. Higher CPC levels (≥0.0101%) were associated with poorer outcomes, suggesting that CPCs may serve as a valuable prognostic marker and could inform treatment stratification in NDMM.

## Dodatak

### Availability of data and materials

The data used to support the findings of this study are available from the corresponding author upon request.

### Funding

No funds, grants, or other support was received.

### Acknowledgements

Not applicable.

### Author contributions

Mulan Jin conceived and designed the study, Hong Chen and Yuan Zhao wrote and revised the manuscript, Zhiyu Zhang and Yan Xie collected and analysed data, Hong Chen and Yuan Zhao made equal contributions to this work as co-first authors. All authors read and approved the final submitted manuscript.

### Conflict of interest statement

All the authors declare that they have no conflict of interest in this work.
